# The prognostic role of the change in albumin-derived neutrophil-to-lymphocyte ratio during neoadjuvant chemoradiotherapy in patients with locally advanced rectal cancer

**DOI:** 10.17305/bb.2023.9787

**Published:** 2024-06-01

**Authors:** Zhen Pan, Ye Wang, Shoufeng Li, Huajun Cai, Guoxian Guan

**Affiliations:** 1 Department of Colorectal Surgery, The First Affiliated Hospital of Fujian Medical University, Fuzhou, China; 2 Department of Colorectal Surgery, National Regional Medical Center, Binhai Campus of the First Affiliated Hospital, Fuzhou, China

**Keywords:** Albumin-derived neutrophil-to-lymphocyte ratio (Alb-dNLR), locally advanced rectal cancer (LARC), neoadjuvant chemoradiotherapy (nCRT), prognosis

## Abstract

The prognosis of patients with locally advanced rectal cancer (LARC) has improved with the adoption of a multidisciplinary treatment approach combining neoadjuvant chemoradiotherapy (nCRT) and total mesorectal excision (TME). Developing real-time, sensitive biomarkers to monitor systemic changes during nCRT is of paramount importance. Although the association between albumin-derived neutrophil-to-lymphocyte ratio (Alb-dNLR) and prognosis in various cancers is established, its prognostic value in LARC patients undergoing nCRT is not well studied. This study enrolled a cohort of 618 LARC patients, stratifying them into two groups according to their change in Alb-dNLR (ΔAlb-dNLR) values, using an optimal cut-off point: a low ΔAlb-dNLR group (≤ 0.90) and a high ΔAlb-dNLR group (>0.90). The prognostic significance of ΔAlb-dNLR was evaluated using a Cox proportional hazards model. The 5-year overall survival (OS) rates were 75.2% in the low ΔAlb-dNLR group (≤0.90) and 85.9% in the high ΔAlb-dNLR group (>0.90) (*P* < 0.001). The 5-year disease-free survival (DFS) rates were 71.2% and 80.6%, respectively (*P* ═ 0.016). Multivariate analyses demonstrated that both ΔAlb-dNLR and pre-Alb-dNLR were independent prognostic factors for OS (*P* ≤ 0.001), while ΔAlb-dNLR was demonstrated as an independent prognostic factor for DFS (*P* ═ 0.016). A predictive nomogram, incorporating the ΔAlb-dNLR subgroup, demonstrated enhanced performance (concordance index [C-index] of 0.720 for OS and 0.690 for DFS) compared to the pre-treatment Alb-dNLR subgroup (C-index of 0.700 for OS and of 0.680 for DFS). Therefore, ΔAlb-dNLR shows significant potential as a usable and prognostic biomarker for predicting OS and DFS in LARC patients undergoing nCRT.

## Introduction

Colorectal cancer (CRC) is a prevalent malignancy and a significant contributor to global cancer-related mortality. Rectal cancers constitute approximately 30%–35% of all CRCs, with nearly half of these cases being identified at an advanced local stage. (i.e., locally advanced rectal cancer [LARC]) [1]. The standard of care for patients with LARC involves a combined modality approach that includes the utilization of fluoropyrimidine-based neoadjuvant chemoradiotherapy (nCRT) followed by total mesorectal surgical excision [2]. The utilization of nCRT has been observed to effectively down-stage LARC, subsequently leading to a reduced incidence of local recurrences in the postoperative period [3, 4]. This approach provides enhanced local tumor control, improved tumor resectability, as well as tumor downsizing and downstaging. However, within the population of patients with LARC, neoadjuvant therapy elicits a diverse range of responses, leading to disparate long-term outcomes. Furthermore, it is imperative to implement adjuvant treatment and surveillance measures in accordance with the prognosis of each individual patient [2, 5, 6]. Hence, the identification of pragmatic and readily attainable indicators assumes a significant role in the stratification and prognosis management of patients diagnosed with LARC.

There is an increasing body of evidence indicating that inflammatory responses play a significant role in facilitating the progression of tumors. The immune system employs mediators to attract immune and inflammatory cells, thereby facilitating the proliferation and sustenance of cancer cells [7]. The derived neutrophil-to-lymphocyte ratio (dNLR) has been identified as a significant independent prognostic factor across multiple cancer types [8]. Furthermore, the importance of dNLR has been substantiated in the context of rectal cancer [9]. Albumin, a commonly employed nutritional marker, has been recognized as a prospective indicator of inflammation. The potential involvement of albumin in conferring resistance to lipid peroxidation in the gastrointestinal mucosal membrane has been proposed [10]. Hence, albumin is widely postulated to be intricately linked to the progression of gastrointestinal cancer, prompting numerous investigations that have primarily concentrated on elucidating its correlation with the susceptibility to gastrointestinal cancer, particularly CRC [11, 12]. The combination of dNLR and Alb, defined as the albumin-derived neutrophil-to-lymphocyte ratio (Alb-dNLR), has been shown to be associated with the prognosis of multiple tumors [13, 14]. The variable ΔAlb-dNLR denotes the alteration in Alb-dNLR levels observed throughout the course of nCRT. According to a recent study conducted by Abe et al. [15], it was demonstrated that ΔAlb-dNLR, serving as an indicator of systemic status during neoadjuvant therapy, exhibited favorable efficacy in predicting overall survival (OS) among patients diagnosed with esophageal squamous cell carcinoma (ESCC). However, the prognostic significance of the Alb-dNLR and ΔAlb-dNLR in patients with LARC who have undergone nCRT remains uncertain.

In this study, we employed the combination of serum albumin and dNLR to create Alb-dNLR, subsequently examining the potential correlation between ΔAlb-dNLR and pre-Alb-dNLR with prognosis in patients diagnosed with LARC. Additionally, we compared the prognostic efficacy of ΔAlb-dNLR with pre-Alb-dNLR.

## Materials and methods

### Patients

A total of 618 patients with LARC who underwent nCRT from November 2011 to August 2019 were included in the study. Inclusion criteria were: rectal cancer with pathological confirmation, blood routine and biochemical examination improved before the surgery, and patients aged 18–80 years. Exclusion criteria were: rectal polyp or adenoma, complicated with acute or chronic inflammatory diseases (like acute upper respiratory tract infection, pneumonia, acute pancreatitis, acute appendicitis, and pyelonephritis), presence of other malignant tumors, incomplete clinical pathological information, and stage IV rectal cancer. The tumor staging was determined through various diagnostic procedures, including physical examination, anoscopy, chest CT, abdominal-pelvic CT, endorectal ultrasound, transrectal ultrasound, and magnetic resonance imaging. Surgical interventions were conducted after a six- to eight-week interval following the completion of radiation treatments. According to the NCCN [16] guidelines, postoperative adjuvant chemotherapy is recommended to commence one month after the surgery.

### Treatment strategy

All patients were administered preoperative radiation therapy at a dose of 45 Gy/25, delivered to the pelvis over a period of five weeks. This was followed by a boost of 5.4-Gy specifically targeting the primary tumor. The preoperative concurrent chemoradiotherapy regimens employed were capecitabine plus oxaliplatin (CAPEOX), capecitabine, and FOLFOX (5FU plus oxaliplatin). The surgical intervention is typically conducted within a timeframe of six to eight weeks following the conclusion of radiation therapy. Middle and low rectal cancers were managed through total mesorectal excision (TME), while high rectal cancers were addressed through partial TME, ensuring a distal margin of 5 cm. Subsequently, patients received postoperative adjuvant chemotherapy approximately four to eight weeks after the surgical procedure, irrespective of the outcomes of the surgical pathology assessment.

### Data collection and definitions

Blood measurements, including white blood cell count, neutrophil count, and albumin levels, were collected at two distinct time points: the initial visit to the doctor and immediately prior to the surgical procedure. Clinicopathological data, encompassing patient characteristics, tumor characteristics, operative details, and postoperative complications, were extracted from the patients’ medical records. Pathological complete response (pCR) is the absence of tumor cells in the primary site and resected lymph nodes. A routine blood test was conducted at the time of the first cancer diagnosis. The calculation of the dNLR (neutrophil count/[leukocyte count − neutrophil count]) involved determining the ratio between the neutrophil count and the difference between the leukocyte count and the neutrophil count, as outlined in a prior study. Similarly, the Alb-dNLR (serum albumin level/dNLR) was obtained by dividing the serum albumin level by the dNLR value. We calculated the Alb-dNLR before nCRT (pre-Alb-dNLR) and after nCRT (post-Alb-dNLR). The change in the Alb-dNLR during nCRT (ΔAlb-dNLR) was calculated by dividing the post-Alb-dNLR by the pre-Alb-dNLR. This study calculated OS from the surgical date to the last follow-up or death date (specifically, cancer-related deaths were not considered). DFS was the survival time until a local or distant disease recurred.

### Ethical statement

This study was approved by the Ethics Committee of The First Affiliated Hospital of Fujian Medical University, the approval number 2021323. We confirm that the study was conducted in accordance with the relevant guidelines/regulations, and all participants and/or their legal guardians provided informed consent. Informed consent for research purposes with patient data and images was obtained for all patients.

**Figure 1.**
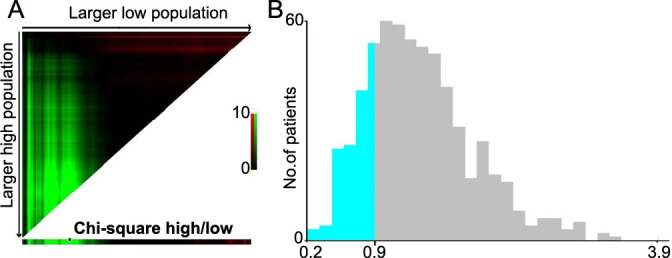
**The determination of cut-off points for ΔAlb-dNLR conducted using the X-tile program.** (A) The X-tile analysis involved the division of the entire cohort into the training sets (displayed in the upper-left quartile) and matched validation sets (displayed along the bottom *X*-axis), utilizing patient survival data. The black dot within the validation set signifies the precise cutoff values for the ΔAlb-dNLR count. (B) Subsequently, the entire cohort was segregated into low (blue) and high (gray) ΔAlb-dNLR count groups based on the optimal cutoff point of 0.90, as depicted in the histogram of the entire cohort. ΔAlb-dNLR: Change in albumin-derived neutrophil-to-lymphocyte ratio before and after neoadjuvant chemotherapy.

### Statistical analysis

Statistical analysis was conducted using the SPSS 22.0 software package and the R statistical package (http://cran.r-project.org/). The X-tile program (http://www.tissuearray.org/rimmlab/) was used to calculate and determine the best cut-off points for the pre-Alb-dNLR and ΔAlb-dNLR counts. The chi-square test or Fisher’s exact test was employed for the analysis of categorical variables, while the analysis of continuous variables involved the use of Student’s *t*-test or the Mann–Whitney *U* test. An analysis of OS results (survival rates) was performed using the Kaplan–Meier (KM) method. A Cox regression model was utilized to determine risk factors for OS and DFS. The nomogram was constructed based on the final predictive model by using the R statistical package with the survival and rms package. The nomogram was validated internally (1000 bootstrap resamples) to correct overfitting. A bootstrapping method is a nonparametric data-generating method in which new datasets are repeatedly generated from an original dataset and created by random drawing from the sample with replacement. The predictive performance of the nomogram was assessed by calculating Harrell’s concordance index (C-index). Nomogram calibration for three- and five-year OS and DFS was performed by comparing the predicted and actual probability after bias correction. *P* < 0.05 was considered statistically significant.

## Results

### Patient characteristics

**Figure S1.**
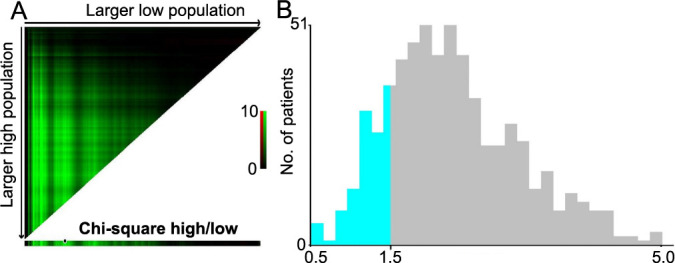
**The determination of cut-off points for Pre-Alb-dNLR conducted using the X-tile program.** Pre-Alb-dNLR: Albumin-derived neutrophil-to-lymphocyte ratio before neoadjuvant chemotherapy.

A total of 618 patients with LARC who underwent nCRT were included in the database, of whom 399 were men and 219 were women. The median follow-up time was 87.3 months for all patients. As seen in Figure 1A and 1B and Figure S1, X-tile plots were identified 0.90 and 1.52 as cut-off points for ΔAlb-dNLR and pre-Alb-dNLR counts, respectively. Based on the above cut-off points, we divided the entire cohort into low (ΔAlb-dNLR ≤ 0.90) and high (ΔAlb-dNLR > 0.90) OS and DFS subgroups. One hundred twenty-two patients (20%) had a low ΔAlb-dNLR (the distribution of ΔAlb-dNLR is shown in Figure 1B). Table 1 summarizes the clinical characteristics of the two groups. A significant difference between the two groups was observed in the occurrence of anemia (*P* ═ 0.034). In the low ΔAlb-dNLR group, the pre-Alb-dNLR was lower (*P* < 0.001), whereas the post-Alb-dNLR was higher than the corresponding scores in the high ΔAlb-dNLR group (*P <* 0.001).

**Table 1:** Clinicopathological characteristics of all patients

**Characteristics**	**ΔAlb-dNLR ≤ 0.90**	**ΔAlb-dNLR > 0.90**	***P* value**
	**(*n* ═ 122)**	**(*n* ═ 496)**	
Age (years), mean (SD)	56.20 ± 11.96	56.15 ± 10.74	0.969
Distance from the anal verge (cm), mean (SD)	6.63 ± 2.89	6.57 ± 2.49	0.808
Interval time between nCRT and surgery (days), mean (SD)	64.48 ± 24.65	65.00 ± 19.71	0.807
Tumor size (cm), mean (SD)	3.91 ± 1.29	3.67 ± 1.27	0.056
Postoperative hospital stay (days), mean (SD)	9.06 ± 5.91	8.53 ± 5.12	0.186
Total hospitalization day (days), mean (SD)	19.73 ± 8.02	19.72 ± 6.37	0.984
Pre-Alb-dNLR level (g/dL), mean (SD)	2.83 ± 0.98	2.20 ± 0.77	**< 0.001**
Post-Alb-dNLR level (g/dL), mean (SD)	1.95 ± 0.76	3.23 ± 1.04	**< 0.001**
Pre-albumin level (g/dL), mean (SD)	4.12 ± 0.29	4.10 ± 0.34	0.420
Post-albumin level (g/dL), mean (SD)	4.05 ± 0.37	4.10 ± 0.31	0.184
Adjuvant chemotherapy, *n* (%)			0.571
No	12 (9.4)	55 (91.7)	
Yes	115 (90.6)	436 (8.3)	
Postoperative complication, *n* (%)			0.557
No	110 (86.6)	415 (84.5)	
Yes	17 (13.4)	76 (15.5)	
Sex, *n* (%)			0.371
Male	83 (68.0)	316 (63.7)	
Female	39 (32.0)	180 (36.3)	
ASA score, *n* (%)			0.531
I	88 (72.1)	372 (75.0)	
II	31 (25.4)	118 (23.8)	
III	3 (2.5)	6 (1.2)	
Surgery approach, *n* (%)			0.562
Laparoscopic	80 (65.6)	337 (67.9)	
Open	28 (23.0)	114 (23.0)	
Robotic	12 (9.8)	43 (8.7)	
Laparoscopic to open	2 (1.6)	2 (0.4)	
ypTNM stage (8th AJCC), *n* (%)			0.247
pCR	36 (29.5)	105 (21.2)	
I	28 (23.0)	129 (26.0)	
II	33 (27.0)	139 (28.0)	
III	25 (20.5)	123 (24.8)	
APR, *n* (%)			0.265
No	108 (88.5)	455 (91.7)	
Yes	14 (11.5)	41 (8.3)	
Neural invasion, *n* (%)			0.585
Yes	6 (4.9)	19 (3.8)	
No	116 (95.1)	477 (96.2)	
Lymphovascular invasion, *n* (%)			0.527
Yes	2 (1.6)	16 (3.2)	
No	120 (98.4)	480 (96.8)	
Radiotherapy complication, *n* (%)			0.394
Yes	34 (27.9)	158 (31.9)	
No	88 (72.1)	338 (68.1)	
TRG grade, *n* (%)			0.205
0	37 (30.3)	108 (21.8)	
1	36 (29.5)	165 (33.3)	
2	41 (33.6)	196 (39.5)	
3	8 (6.6)	27 (5.4)	
Anemia, *n* (%)			**0.034**
Yes	27 (22.1)	71 (14.3)	
No	95 (77.9)	425 (85.7)	
Chemotherapy, *n* (%)			0.462
CapeOX	21 (16.5)	74 (15.1)	
Capecitabine	87 (68.5)	361 (73.5)	
FOLFOX	19 (15.0)	56 (11.4)	

Bold values indicate statistical significance. dNLR: Derived neutrophil-to-lymphocyte ratio; Alb: Serum albumin value; ΔAlb-dNLR: Change in albumin-derived neutrophil-to-lymphocyte ratio before and after neoadjuvant chemotherapy; Pre-Alb-dNLR level: Albumin-derived neutrophil-to-lymphocyte ratio before neoadjuvant chemotherapy; Post-Alb-dNLR level: Albumin-derived neutrophil-to-lymphocyte ratio after neoadjuvant chemotherapy; Pre-albumin level: Albumin before neoadjuvant chemotherapy; Post-albumin level: Albumin after neoadjuvant chemotherapy; nCRT: Neoadjuvant chemoradiotherapy; ASA: American Society of Anesthesiologists; TRG grade: Tumor regression grade; APR: Abdominoperineal resection; SD: Standard deviation; CapeOX: Capecitabine and oxaliplatin; FOLFOX: Folinic acid, fluorouracil, and oxaliplatin; ypTNM: Yield pathological tumor node metastasis stage; AJCC: American Joint Committee on Cancer.

### Association of ΔAlb-dNLR with survival

**Figure 2.**
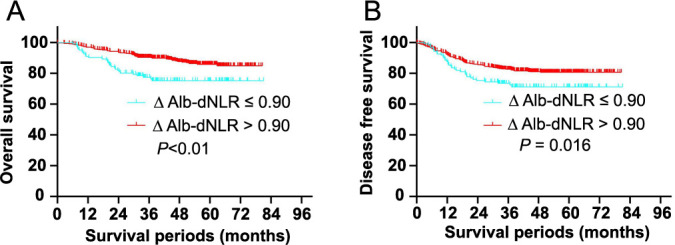
**Kaplan–Meier analysis of the ΔAlb-dNLR counts.** The overall survival (A) and disease-free survival (B) for the optimal cutoff point of the ΔAlb-dNLR counts. The entire cohort was divided into low (blue) and high (red) ΔAlb-dNLR count groups based on the optimal cutoff point (0.90). ΔAlb-dNLR: Change in albumin-derived neutrophil-to-lymphocyte ratio before and after neoadjuvant chemotherapy.

The optimal cut-off point of ΔAlb-dNLR was conducted based on X-tile software (X-tile 3.6.1). To categorize our cohort according to the above cut-off points, we divided them into low and high subgroups in OS and DFS. Low ΔAlb-dNLR LARC patients had a worse prognosis during nCRT. In the high ΔAlb-dNLR groups, OS rates at five years were 85.9%, significantly higher than 75.2% in the low ΔAlb-dNLR groups (*P* < 0.001, Figure 2A). Notably, a significant association exists between higher ΔAlb-dNLR scores and better DFS. DFS rates at five years for the high ΔAlb-dNLR group are 80.6%, compared to 71.2% for the low ΔAlb-dNLR group (*P* ═ 0.016, Figure 2B).

### Association of ΔAlb-dNLR with prognostic significance of OS and DFS

In order to investigate the prognostic significance of ΔAlb-dNLR on OS and DFS in patients with LARC, a Cox regression analysis was conducted. In the univariate analysis, several factors including the long diameters of the tumor (*P* < 0.001), pathological TNM stage (*P* < 0.001), TRG grade (*P* < 0.001), neural invasion (*P* ═ 0.003), and ΔAlb-dNLR level (*P* ═ 0.001) were found to be independently associated with OS in patients with LARC who underwent nCRT and TME (Table 2). The Cox regression analysis revealed that the pathological TNM stage (*P* ═ 0.007), the long diameters of the tumor (hazard ratio [HR] ═ 1.174, 95% confidence interval [CI] 1.006–1.371, *P* ═ 0.042), the pre-Alb-dNLR level (HR ═ 0.462, 95%CI 0.283–0.752, *P* ═ 0.002), and ΔAlb-dNLR level (HR ═ 0.404, 95%CI 0.255–0.639, *P* < 0.001) were identified as significant independent predictors of OS following nCRT, as presented in Table 2.

**Table 2:** Univariate and multivariate analysis of overall survival

**Variable**	**Univariate analysis**		**Multivariate analysis**	
	**HR (95%CI)**	***P* value**	**HR (95%CI)**	***P* value**
Age (years)	0.997 (0.978 – 1.016)	0.716		
Distance from the anal verge (cm)	0.949 (0.866 – 1.040)	0.262		
Interval time between nCRT and surgery (days)	0.993 (0.976 – 1.009)	0.375		
Total hospitalization days (days)	1.010 (0.982 – 1.039)	0.492		
Postoperative hospital stay (days)	1.004 (0.964 – 1.046)	0.844		
Surgical time (min)	1.003 (1.000 – 1.006)	0.054		
Intraoperative bleeding (mL)	1.002 (1.000 – 1.003)	0.080		
Tumor size (cm)	1.354 (1.178 – 1.556)	**<0.001**	1.174 (1.006 – 1.371)	**0.042**
Adjuvant chemotherapy		0.467		
No	1			
Yes	0.782 (0.403 – 1.518)			
Postoperative complication		0.633		
No	1			
Yes	0.856 (0.454 – 1.616)			
Sex		0.923		
Female	1			
Male	0.979 (0.636 – 1.507)			
ASA score		0.838		
I	1			
II	1.132 (0.709 – 1.808)	0.603		
III	0.774 (0.107 – 5.578)	0.799		
ypTNM stage (8th AJCC)		**<0.001**		**0.007**
pCR	1		1	
I	1.294 (0.553 – 3.028)	0.552	0.770 (0.073 – 8.161)	0.828
II	2.610 (1.227 – 5.550)	**0.013**	1.138 (0.113 – 11.495)	0.913
III	4.979 (2.419 – 10.247)	**<0.001**	2.123 (0.218 – 20.651)	0.517
ΔAlb-dNLR level		**0.001**		**<0.001**
≤0.90	1		1	
>0.90	0.461 (0.296 – 0.718)		0.404 (0.255 – 0.639)	
Pre-Alb-dNLR level (g/dL)		**0.004**		**0.002**
≤1.52	1			
>1.52	0.508 (0.320 – 0.805)		0.462 (0.283 – 0.752)	
APR		0.633		
No	1			
Yes	1.183 (0.594 – 2.355)			
Radiotherapy complication		0.463		
No	1			
Yes	1.176 (0.763 – 1.813)			
Neural invasion		**0.003**		0.393
No	1		1	
Yes	2.998 (1.445 – 6.219)		1.409 (0.641 – 3.097)	
Lymphovascular invasion		0.682		
No	1			
Yes	1.272 (0.402 – 4.020)			
TRG grade		**<0.001**		0.060
0	1		1	
1	1.790 (0.824 – 3.888)	0.141	1.405 (0.136 – 14.511)	0.775
2	3.466 (1.699 – 7.073)	**0.001**	2.508 (0.249 – 25.219)	0.435
3	6.793 (2.862 – 16.127)	**<0.001**	3.342 (0.329 – 33.939)	0.308
Anemia		0.231		
No	1			
Yes	0.729 (0.435 – 1.222)			

Bold values indicate statistical significance. ΔAlb-dNLR: Change in albumin-derived neutrophil-to-lymphocyte ratio before and after neoadjuvant chemotherapy; Pre-Alb-dNLR level: Albumin-derived neutrophil-to-lymphocyte ratio before neoadjuvant chemotherapy; ASA: American Society of Anesthesiologists; TRG grade: Tumor regression grade; APR: Abdominoperineal resection; CI: Confidence interval; HR: Hazard ratio; ypTNM: Yield pathological tumor node metastasis stage; AJCC: American Joint Committee on Cancer.

In the conducted analysis, several factors were found to be independently associated with DFS in patients with LARC who underwent nCRT and TME. These factors included the long diameters of tumor (*P* < 0.001), pathological TNM stage (*P* < 0.001), TRG grade (*P* < 0.001), distance from the anal verge (*P* ═ 0.012), ΔAlb-dNLR level (*P* ═ 0.017), and neural invasion (*P* ═ 0.025), as shown in Table 3. The results of the Cox regression analysis revealed that the pathological TNM stage (*P* ═ 0.005), the long diameters of tumor (HR ═ 1.1193, 95%CI 1.043–1.364, *P* ═ 0.010), ΔAlb-dNLR level (HR ═ 0.642, 95%CI 0.430–0.959, *P* ═ 0.030), and distance from the anal verge (HR ═ 0.902, 95%CI 0.835–0.975, *P* ═ 0.009) were identified as independent predictors of DFS after nCRT, as shown in Table 3.

### Development and validation of the nomogram

**Figure 3.**
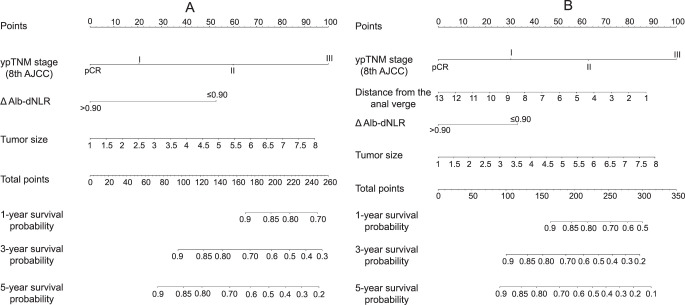
**Construction of the factors for overall survival/disease-free survival.** Nomograms developed for predicting (A) overall survival and (B) disease-free survival. ΔAlb-dNLR: Change in albumin-derived neutrophil-to-lymphocyte ratio before and after neoadjuvant chemotherapy; ypTNM: Yield pathological tumor node metastasis stage; AJCC: American Joint Committee on Cancer.

**Figure 4.**
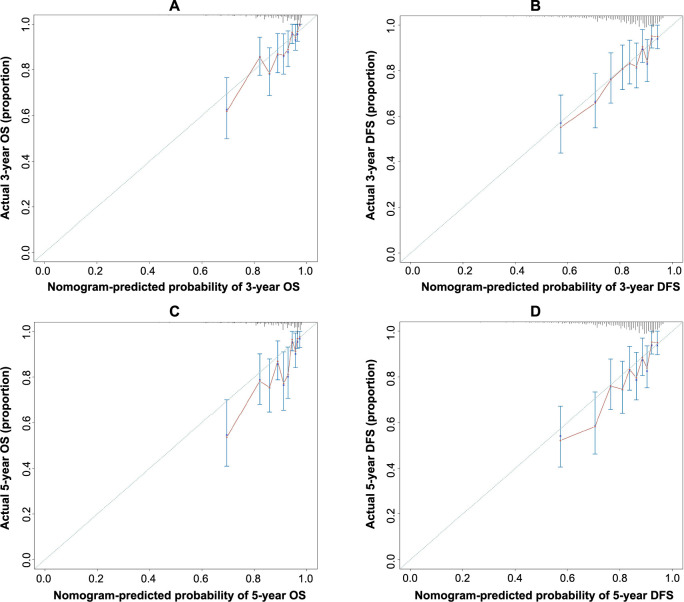
**Calibration curves.** (A and C) For 3- and 5-year OS for the model with ΔAlb-dNLR counts in LARC patients after nCRT with internal validation; (B and D) For 3- and 5-year DFS for the model with ΔAlb-dNLR counts in LARC patients after nCRT with internal validation. OS: Overall survival; ΔAlb-dNLR: Change in the albumin-derived neutrophil-to-lymphocyte ratio during neoadjuvant chemoradiotherapy; LARC: Locally advanced rectal cancer; nCRT: Neoadjuvant chemoradiotherapy; DFS: Disease-free survival.

**Figure S2.**
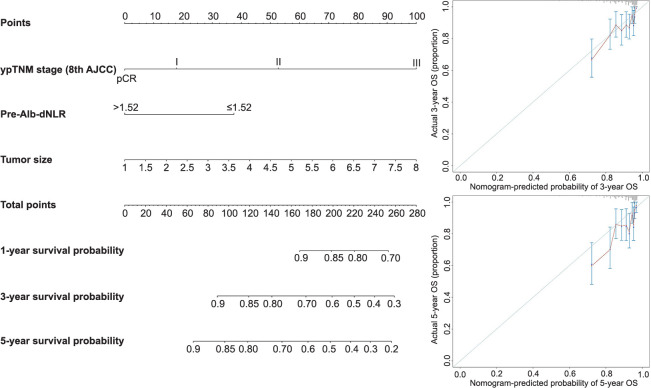
**Nomogram construction based on Pre-Alb-dNLR.** Pre-Alb-dNLR: Albumin-derived neutrophil-to-lymphocyte ratio before neoadjuvant chemotherapy.

Predictive nomograms for OS and DFS in patients with LARC following nCRT were developed based on the aforementioned significant determinants (Figure 3A and 3B). The predictive probabilities for three-year OS and disease-free survival (DFS) were determined by summing the scores of each variable and plotting a linear regression line. Patients with higher total scores exhibited a tendency toward lower rates of OS and DFS. The internal validation of the model demonstrated its performance. The C-index of the nomogram, which incorporated ΔAlb-dNLR as a predictor for OS and DFS, was calculated as 0.720 (95%CI 0.696–0.744) and 0.690 (95%CI 0.667–0.713), respectively. To further explore the role of the ΔAlb-dNLR in the predictive model, we constructed another model with Pre-Alb-dNLR (Figure S2). The C-index of the nomogram with Pre-Alb-dNLR for predicting OS and DFS was 0.700 (95%CI 0.687–0.726) and 0.680 (95%CI 0.657–0.703), respectively. The calibration curves showed good agreement between the predicted and actual probability of three- and five-year OS (Figure 4A and 4C) and DFS (Figure 4B and 4D).

**Figure 5.**
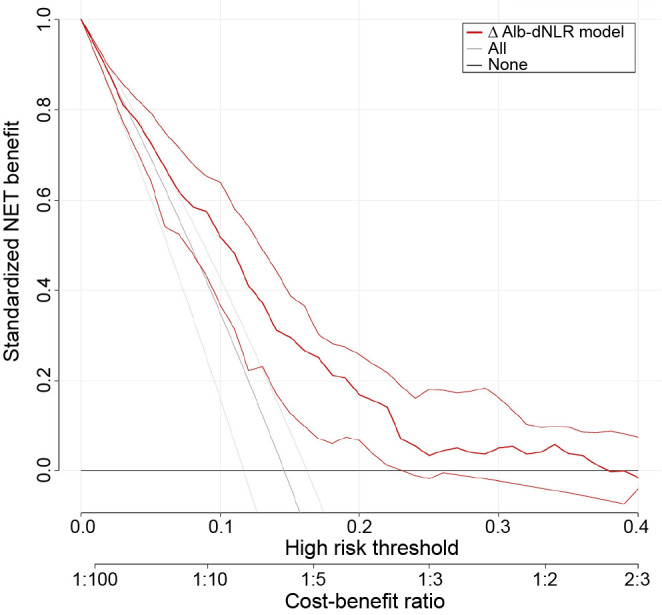
**Decision curve analysis curve of model ΔAlb-dNLR.** ΔAlb-dNLR: Change in the albumin-derived neutrophil-to-lymphocyte ratio during neoadjuvant chemoradiotherapy.

Furthermore, a decision curve analysis (DCA) was performed, employing the net benefit rate as an ordinal measure, while setting the high-risk threshold to a negative value of (0.1) (Figure 5). As depicted in Figure 5, the net benefit rate exceeded 0 within the high-risk threshold range of 0–1, indicating clinical significance.

## Discussion

Prior research has demonstrated a strong correlation between Alb-dNLR and the advancement of cancer as well as its prognosis. However, the role of Alb-dNLR in LARC receiving nCRT remains unclear. This study aimed to examine the significance of ΔAlb-dNLR in predicting the prognosis of LARC patients undergoing neoadjuvant therapy. To the best of our knowledge, this is the inaugural investigation utilizing ΔAlb-dNLR as a prognostic indicator for LARC. This study demonstrated that the ΔAlb-dNLR variable functioned as an independent prognostic factor for LARC following nCRT, surpassing the pre-Alb-dNLR variable in predictive accuracy. Furthermore, a prognostic nomogram was developed by incorporating ΔAlb-dNLR, and its performance was assessed through DCA and calibration curve analysis.

Numerous studies have amassed evidence indicating a strong association between systemic inflammation and the advancement and prognostication of cancer [17]. Neutrophils and lymphocytes played crucial roles in facilitating the carcinogenesis and progression of LARC within the context of tumor-related inflammation. Neutrophils recruited and subsequently released reactive oxygen species in order to facilitate the induction of DNA damage and genetic instability within rectal epithelial cells [18]. Moreover, it secreted VEGF, ANGPT1, and FGF-2 to facilitate angiogenesis and tumor invasion [19]. IL-6, TNF-α, and granulocyte-colony-stimulating factor (G-CSF) have been identified as significant contributors to neutrophilia in various malignancies, including anal cancer, cervical cancer, and CRC [20–23]. On the other hand, infiltrating lymphocytes typically exerted an antitumor effect through the adaptive immune response. Specifically, T-cells, including CD4^+^ T cells and CD8^+^ T cells, played a crucial role in regulating the host’s immune response to CRC [24]. CD4^+^ Th1 cells, in particular, were capable of releasing IL-2 to enhance the cytotoxicity of CD8^+^ T cells. Additionally, these Th1 cells could directly impede cancer cell proliferation by secreting IFN-γ and TNF [25, 26]. Neutrophils were found to inhibit the cytolytic activity of T cells during an inflammatory reaction [27]. The neutrophil-to-lymphocyte ratio (NLR) is an inflammatory index that has been linked to the prognosis of cancer patients [28]. Prior studies have demonstrated the association between NLR and both prognosis and treatment response in LARC as well as other malignancies [29–32]. The calculation of the NLR can be readily performed using laboratory parameters, specifically neutrophil counts and lymphocyte counts, which are routinely obtained prior to surgical procedures. The initial documentation of the dNLR was conducted by Proctor et al. [33], who demonstrated that both the NLR and dNLR possess comparable prognostic significance across various cancer types. The NLR is determined by dividing the neutrophil count by the lymphocyte count, while the dNLR can be calculated using the total white blood cell count and neutrophil count without the need for lymphocytes. Consequently, the dNLR offers a simpler and more convenient approach. Subsequent studies have demonstrated the potential prognostic value of dNLR in various types of cancer, including ovarian cancer [34], breast cancer [35], and pancreatic cancer (PCC) [36].

**Table 3:** Univariate and multivariate analysis of disease-free survival

**Variable**	**Univariate analysis**		**Multivariate analysis**	
	**HR (95%CI)**	***P* value**	**HR (95%CI)**	***P* value**
Age (years)	0.995 (0.979 – 1.011)	0.521		
Distance from the anal verge (cm)	0.903 (0.835 – 0.978)	**0.012**	0.902 (0.835 – 0.975)	**0.009**
Interval time between nCRT and surgery (days)	0.995 (0.983 – 1.007)	0.450		
Total hospitalization days (days)	1.015 (0.992 – 1.038)	0.196		
Postoperative hospital stay (days)	1.014 (0.984 – 1.045)	0.362		
Surgical time (min)	1.002 (0.999 – 1.055)	0.129		
Intraoperative bleeding (mL)	1.001 (0.999 – 1.003)	0.354		
Tumor size (cm)	1.290 (1.142 – 1.457)	**<0.001**	1.193 (1.043 – 1.364)	**0.010**
Adjuvant chemotherapy		0.864		
No	1			
Yes	0.951 (0.535 – 1.691)			
Postoperative complication		0.890		
No	1			
Yes	1.035 (0.634 – 1.689)			
Sex		0.264		
Female	1			
Male	0.816 (0.571 – 1.166)			
ASA score		0.972		
I	1			
II	1.029 (0.680 – 1.557)	0.891		
III	1.159 (0.285 – 4.709)	0.836		
ypTNM stage (8th AJCC)		**<0.001**		**0.005**
pCR	1		1	
I	1.459 (0.747 – 2.851)	0.269	1.327 (0.201 – 8.749)	768
II	2.347 (1.269 – 4.341)	**0.007**	1.867 (0.290 – 12.029)	0.511
III	4.352 (2.414 – 7.854)	**<0.001**	3.056 (0.494 – 18.892)	0.229
ΔAlb-dNLR level		**0.017**		**0.030**
≤0.90	1		1	
>0.90	0.618 (0.417 – 0.916)		0.642 (0.430 – 0.959)	
Pre-Alb-dNLR level (g/dL)		0.122		
≤1.52	1			
>1.52	0.715 (0.467 – 1.092)			
Anal-preserving		0.166		
No	1			
Yes	1.464 (0.854 – 2.511)			
Radiotherapy complication		0.075		
No	1			
Yes	1.387 (0.968 – 1.988)			
Neural invasion		**0.025**		0.950
No	1		1	
Yes	2.175 (1.103 – 4.290)		0.977 (0.472 – 2.025)	

Bold values indicate statistical significance. ΔAlb-dNLR: Change in albumin-derived neutrophil-to-lymphocyte ratio before and after neoadjuvant chemotherapy; Pre-Alb-dNLR level: Albumin-derived neutrophil-to-lymphocyte ratio before neoadjuvant chemotherapy; nCRT: Neoadjuvant chemoradiotherapy; ASA: American Society of Anesthesiologists; CI: Confidence interval; HR: Hazard ratio; ypTNM: Yield pathological tumor node metastasis stage; AJCC: American Joint Committee on Cancer.

Albumin, a widely used nutritional marker, has been identified as a potential indicator of inflammation. Previous studies have demonstrated the utility of serum albumin levels as a predictive factor for both nutritional status and inflammatory response in cancer patients [37, 38]. Albumin, known for its selective accumulation in tumor tissue, serves as a significant source of energy and nutrition for the proliferation of tumors. Moreover, the presence of hypoalbuminemia can detrimentally affect cellular immunity, ultimately leading to an unfavorable prognosis. Al-Shaiba et al. [39] have substantiated a correlation between hypoalbuminemia, inflammation, and unfavorable prognosis among individuals diagnosed with colorectal liver metastases. Therefore, it is noteworthy that albumin concentration serves as both a nutritional marker and an indicator of inflammatory response. A growing body of evidence suggests that hypoalbuminemia is linked to unfavorable survival outcomes in various primary malignancies, including LARC [40–42].

The Alb-dNLR score, an index that integrates the nutritional index Alb and the inflammation index dNLR, has emerged as a recent and straightforward tool. Chen et al. [43] have demonstrated the association between the Alb-dNLR score and DAS, a metric for assessing rheumatoid arthritis activity, as well as inflammatory biomarkers including C-reactive protein, erythrocyte sedimentation rate, and IgA. Additionally, it was demonstrated that the utilization of both albumin and dNLR in conjunction may yield superior results in terms of diagnostic efficacy for rheumatoid arthritis. Furthermore, the combination of circulating dNLR and Alb demonstrated a significant association with the survival of individuals with PCC and exhibited potential for enhancing the diagnostic accuracy of PCC [13]. In another investigation conducted by Sun et al. [14], a comparison was made between Alb-dNLR and other prognostic indexes through the construction of ROC curves. The findings demonstrated that Alb-dNLR exhibited superior predictive performance in relation to other prognostic indicators. Consequently, it can be utilized as a reliable tool for forecasting postoperative OS in individuals diagnosed with gastric cancer, thereby facilitating the implementation of targeted therapeutic interventions. These findings suggest that Alb-dNLR is strongly associated with cancer progression and prognosis.

However, it is noteworthy that the majority of studies have primarily concentrated on pre-nCRT conditions [44–46]. One drawback is their inability to consider the post-neoadjuvant chemotherapy (nCT) status. The alteration in systemic inflammation caused by nCRT had an impact on the prognosis and treatment response of patients with LARC who underwent nCRT followed by surgery. Therefore, it is imperative to develop real-time and sensitive biomarkers that can effectively track alterations in systemic conditions during nCRT, as they play a crucial role in formulating treatment strategies tailored to individual risk levels and ensuring continuous patient monitoring. ΔAlb-dNLR serves as a reliable measure to assess the dynamic alterations occurring during neoadjuvant therapy, enabling real-time monitoring of both the inflammatory response and nutritional resistance. In a recent study conducted by Abe et al. [15], it was demonstrated that ΔAlb-dNLR serves as a convenient and valuable prognostic indicator for OS in patients with ESCC who undergo nCT. Furthermore, the study findings indicate that ΔAlb-dNLR exhibits superior predictive efficacy compared to pre-Alb-dNLR. Therefore, we speculated that ΔAlb-dNLR might be a promising tool in predicting outcomes in LARC. We conducted an assessment on the utilization of pre-Alb-dNLR and ΔAlb-dNLR among patients diagnosed with LARC and compared their respective predictive effectiveness. In the current study, we found that the ΔAlb-dNLR was a valuable predictor of OS and DFS independent of the TNM stage. Furthermore, it was significantly associated with some variables indicating poor prognosis, including higher TNM stage and larger tumor size. Obviously, an elevated ΔAlb-dNLR score was associated with more aggressive tumor features. In the model performance analyses, the predictive Nomogram, which included the ΔAlb-dNLR subgroup (OS C-index – 0.720, DFS – 0.690), exhibited superior performance compared to those including the pre-Alb-dNLR subgroup (OS C-index – 0.700, DFS – 0.680).

In the present study, it was observed that the ΔAlb-dNLR parameter exhibits a significant correlation with the prognosis of patients diagnosed with LARC who underwent nCRT. Notably, the calculation of ΔAlb-dNLR relies on commonly employed clinical indices that are routinely utilized in everyday clinical practice. Subsequently, we conducted an assessment on the utilization of ΔAlb-dNLR among patients diagnosed with LARC. The findings indicated that a reduction of less than 90% in Alb-dNLR was linked to an unfavorable prognosis in LARC patients. This outcome holds significance as the decline in Alb-dNLR proves to be a more robust prognostic determinant compared to pre-Alb-dNLR. The decreased Alb-dNLR during nCRT suggests a decrease in albumin levels and/or an increase in dNLR levels. Both of these responses indicate tumor progression during nCRT, although the exact mechanism is not fully understood. It has been reported that this phenomenon may be associated with inflammatory response and nutritional resistance during neoadjuvant therapy.

The utilization of the preoperative ΔAlb-dNLR score has the potential to discern patients with an unfavorable prognosis. In such cases, it would be advantageous to allocate resources more effectively toward those patients with a higher risk, including implementing more frequent monitoring and administering more intensive adjuvant chemotherapy. In addition, such patients may benefit from targeted anti-inflammatory therapy after surgery [47]. Furthermore, the utilization of certain anti-inflammatory agents could potentially be guided by the ΔAlb-dNLR score. Finally, it is worth investigating whether implementing a targeted preoperative nutritional intervention or neoadjuvant therapy could enhance outcomes in patients with a ΔAlb-dNLR score reduction of less than 90% during nCRT.

Some limitations were present in our study. Since this was a retrospective single-center investigation, we require a prospective study design to evaluate our findings. Second, our sample size was limited. Thus, multicenter studies could provide a larger sample size for further investigation.

## Conclusion

The ΔAlb-dNLR demonstrates high usability and prognostic value in predicting OS and DFS outcomes among patients diagnosed with LARC who undergo nCRT.

## Supplemental data

## Data Availability

Some or all data used during the study are available from the corresponding author by request.
